# The Autonomic Regulation of Tumor Growth and the Missing Links

**DOI:** 10.3389/fonc.2020.00744

**Published:** 2020-05-13

**Authors:** Maricris Bautista, Anand Krishnan

**Affiliations:** ^1^Department of Anatomy, Physiology, and Pharmacology, College of Medicine, University of Saskatchewan, Saskatoon, SK, Canada; ^2^Cameco MS Neuroscience Research Centre (CMSNRC), University of Saskatchewan, Saskatoon, SK, Canada

**Keywords:** nerve-tumor interface, nerve-tumor crosstalk, nerve-dependence of cancers, metastasis, norepinephrine, acetylcholine, neurotrophic factors

## Abstract

Accumulating evidence now indicates that peripheral nerves and solid tumors mutually support the growth of each other. Tumor-derived molecular cues guide nerve infiltration to the tumor milieu, while the tumor-infiltrating nerves provide molecular support to promote tumor growth and dissemination. In this mini-review, we discuss the unique roles of sympathetic and parasympathetic nerves in promoting tumor growth and metastasis. The contribution of adrenergic and cholinergic signals, the specific receptors involved, and the downstream molecular links in both cancer cells and stromal cells are discussed for their intrinsic capacity to modulate tumor growth. We identified unappreciated niche areas in the field, an investigation of which are critical to filling the knowledge gap in understanding the biology of neuromodulation of cancers.

## Introduction

The tumor microenvironment significantly influences the progression of solid tumors (1). Therefore, there has been a long-standing interest in understanding the functions of stromal cells in the tumor milieu. An immense interest has been recently developed in understanding the functions of peripheral nerves in the tumor microenvironment. For many decades, nerves were only recognized as pain carriers of tumors. However, recent studies demonstrated that peripheral nerves modulate tumor growth and dissemination. The widely recognized belief now is that the tumors attract nerves by stimulating nerve growth, and in turn, the nerves feed both cancer and stromal cells in the tumor milieu (2, 3).

The nerve-derived molecules supporting tumor growth have been reviewed in much detail (2, 3). In this regard, the neurotransmitters released from autonomic nerves have gained much attention, and clinical trials are now underway by blocking the corresponding receptors to managing a variety of solid tumors. Here, we review the nerve-tumor interface with particular attention to the unique contribution of autonomic signaling to tumor growth and dissemination. We also discuss the missing links in the current state of knowledge in understanding the biology of neuromodulation of cancers.

## The Nerve-Tumor Crosstalk: The Milestones

The nerve-dependence of tumors received initial attention due to the occurrence of perineural invasion (PNI), in which cancer cells migrate around and invade nerves (4–6). Although PNI was recognized much earlier as a complex physical interaction between cancer cells and nerves, a mutual growth stimulatory interaction between the cancer cells and neurons was experimentally demonstrated within the past two decades. In an elegant study, Ayala et al. showed that the outgrowth and directionality of sensory neurons and the proliferation and migration of prostate cancer cells were mutually benefitted when they were cultured together (7). This observation kickstarted serious investigations to understanding the nerve-tumor interface.

In the early 2000, clinicians also found an association between underlying stress and poor prognosis of cancer patients (8). Supporting this association, a study by Thaker et al. showed that stress-activated adrenergic signals promote tumor growth, signifying the critical involvement of sympathetic nerves in tumor progression (9). Another breakthrough occurred in 2013 when Magnon et al. demonstrated that adrenergic signals in stromal cells are indeed essential for tumorigenesis (10). The tumor promoting roles of cholinergic signals were also established around this time when it was discovered that cholinergic muscarinic receptors facilitate tumorigenesis and metastasis (10, 11). These discoveries then fuelled a broader interest in understanding the roles of autonomic signals in tumor microenvironment.

## Sympathetic Distribution in the Tumor Microenvironment

Sympathetic nerve innervation in tumors has been demonstrated in prostate, ovarian, and breast tumors by specifically staining the adrenergic neurons with tyrosine hydroxylase (10, 12–14). In addition, animal tumor models of the prostate, melanoma and ovarian cancers showed increased levels of the sympathetic neurotransmitter norepinephrine (NE), indicating that sympathetic activity is enhanced in solid tumors (12, 13, 15). NE has also been implicated in stress-mediated melanoma and ovarian tumor progression (13, 16). The specific receptors for NE, the adrenergic β receptors (Adrβ), are widely distributed in both cancer and stromal cells. For example, the Adrβ are expressed in the prostate, melanoma, ovarian, pancreatic, colon and breast cancer cells, and pericytes, endothelial cells, lymphocytes, and myeloid cells (10, 12–14, 17–22). Among the Adrβ, varying expressions and levels of Adrβ1/ β2/ β3 are reported in several cancer types (23).

## Adrenergic Signals as Tumor Promoters: Molecular Mechanisms

Several growth signaling cascades are activated downstream of Adrβ. For example, Adrβ2 activation was shown to trigger Src kinase to promote ovarian cancer cell proliferation, migration, and invasion (19). A positive association between NE and Src activation was also demonstrated in human ovarian tumors substantiating the tumor promoting roles of the NE-Src axis (19). Adrβ2 also enables cancer cells to escape chemotherapy-induced cytotoxicity. For instance, Adrβ2 signals have shown to activate the survival phosphatase, DUSP1, which in turn dephosphorylates JNK and c-jun to promote ovarian cancer cell survival pre-treated with cisplatin or paclitaxel (24). Thaker et al. showed that activation of Adrβ2 by NE induces VEGF in ovarian cancer cells (9). The VEGF, in turn, signals endothelial cells to facilitate angiogenesis, which is an example of how NE promotes tumor angiogenesis indirectly (9). Interestingly, NE dependent activation of Adrβ3 in ovarian cancer cells induces BDNF through cAMP/JNK activation (13). The BDNF then signals TrKB receptors in the nearby nerves to promote axonogenesis, which is an example of how NE promotes tumor axonogenesis indirectly. A positive association between NE and BDNF was also observed in human ovarian carcinoma, further indicating that the NE-BDNF axis promotes tumors (13).

The tumor-promoting roles of Adrβ3, and the mechanisms involved, have been extensively studied in melanoma. In line with this, Dal Monte et al. showed that blockade of Adrβ3 induces apoptosis of melanoma cells through downregulation iNOS (inducible nitric oxide synthase) mediated NO synthesis (25, 26). Calvani et al. showed that various cellular stresses, such as hypoxia, ischemia, or glucose deprivation induce Adrβ3 in melanoma cells, suggesting that these natural triggers may drive higher production of Adrβ3 in the tumor milieu (21). Strikingly, Adrβ3 promotes the classical Warburg effect (preferred glycolysis) in melanoma stem cells by upregulating UCP2 (uncoupling protein 2) (27). Adrβ3 was also shown to regulate the stemness of neuroblastoma cells wherein Adrβ3 inhibition promoted their differentiation by disrupting the sphingosine kinase 2(sk2)-sphingosine-1-phosphatase receptor 2 (S1P2) axis, which is a lipid metabolic axis otherwise crucial for these cells' stemness and proliferation (28).

The Adrβ have stromal cell-specific actions too. Selective depletion of Adrβ2 and/or Adrβ3 in the stromal compartment prevented the occurrence and early growth of prostate cancer, indicating that stromal cell-specific Adrβ is essential for maturation of oncogenic signals (10). Adrβ2 also facilitates aerobic glycolysis in endothelial cells and promote tumor angiogenesis (12). Both CD4^+^ and CD8^+^ T lymphocytes express Adrβ2, while sympathetic deprivation of these cells downregulates the immune checkpoint protein PD-1 and favor better immune surveillance of breast cancer (14). The Adrβ3 is also expressed in stromal cells, especially fibroblasts, endothelial cells, and immune cells and support tumors and angiogenesis (21, 29). Specific blockade of Adrβ3 was shown to impair endothelial cell survival in melanoma (25). Besides, blockade of Adrβ3 was shown to accentuate the levels of cytotoxic T lymphocytes and natural killer (NK) cells and attenuate the levels of tumor favoring regulatory T cells (Tregs) and myeloid-derived suppressor cells (MDSCs) in melanoma, promoting a favorable immune surveillance (29). Interestingly, Adrβ3 inhibition in myeloid cells switches macrophage and neutrophil phenotypes to immunocompetent M1 and N1 types, respectively, indicating additional mechanisms of Adrβ3 blockers in checking tumors (29). Adrβ3 signals also enrich stromal population by recruiting and maintaining hemopoietic (HSC) and mesenchymal stem cells (MSC) favoring tumor aggression, while Adrβ3 blockade was shown to promote local differentiation of HSC to lymphoid/myeloid lineages and MSC to adipocyte lineages in melanoma favoring a less aggressive tumor milieu (30).

The adrenergic signals also promote migration and invasion of cancer cells. Activation of Adrβ in ovarian and pancreatic cancer cells upregulates MMP-2, MMP-9 and VEGF to facilitate cell migration and invasion (18, 31). A phase II trial showed that a combination of propranolol (a non-specific Adrβ blocker) and etodolac (a non-steroidal anti-inflammatory drug) reduces the levels of epithelial-mesenchymal transition (EMT) genes involved with breast cancer metastasis, suggesting a potential clinical benefit of using β-blockers in fighting metastasis (32). This trial also showed immune profile changes in the treatment cohort (32). However, due to the combined treatment protocol used in this trial, the exact contribution of β-blockers in generating this gene profile is not known. Nonetheless, retrospective analyses showed that β-blockers slow down the progression of multiple myeloma, prostate, melanoma, lung, and ovarian cancers, indicating an apparent additional benefit of targeting adrenergic signals for prolonging disease-free survival (33–37).

**Figure 1:**
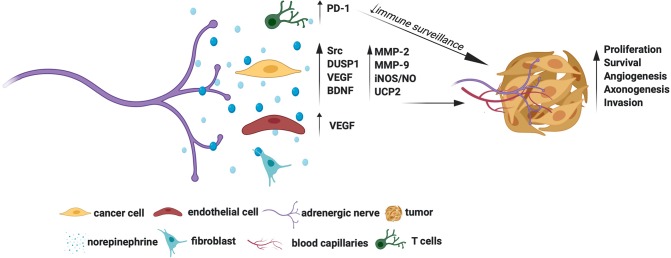
Molecular mechanisms for the tumor promoting actions of adrenergic signals.

Overall, Adrβ receptors, especially the Adrβ2 and Adrβ3, show promise as cancer therapeutic targets. The molecular mechanisms by which adrenergic signals promote tumor growth are summarized in Figure 1. However, it remains to be established whether targeting Adrβ is beneficial in treating all cancer types. For example, inhibition of Adrβ2 showed no effect in gastric tumors (38). Moreover, although NE levels were shown to increase in animal tumors, such an increase in its levels was established only in human ovarian carcinoma (13). Quantification of NE in other tumor types also is warranted to identify its general activation profile in human cancers. Similarly, although Adrβ3 was shown to maintain stem cell traits in tumor milieu, more understanding is required to elucidate the responsible mechanisms.

## Parasympathetic Distribution in the Tumor Microenvironment

In contrast to sympathetic signals, the parasympathetic signals offer both tumor suppressing and promoting functions. The parasympathetic innervation has been demonstrated in gastric, prostate and breast cancers using choline acetyltransferase (ChaT) or vesicular acetylcholine transporter (VAchT) as specific markers (10, 14, 38). The specific receptors of the parasympathetic neurotransmitter acetylcholine, the cholinergic muscarinic receptors (Chrms), are shown to express in gastric, pancreatic, lung, cervical, and colon cancer cells (38–42). Interestingly, lung, pancreatic, cervical and colon cancer cells, and gastric epithelial tuft cells express acetylcholine, independent of nerves (38, 40, 43).

## Cholinergic Signals as Tumor Suppressors: Molecular Mechanisms

Early studies in the ‘80s showed that cholinergic deprivation resulting from vagotomy facilitates gastric tumorigenesis, suggesting that cholinergic signals are essential for tumor suppression (44, 45). Similarly, a recent study by Renz et al. showed that vagotomy promotes pancreatic tumor progression whereas selective activation of Chrm1 reduced tumor incidence (42). Using RNA sequencing studies, the authors showed that Chrm1 signals perturb EGFR/MAPK and PI3K/AKT cascade in cancer cells to inhibit tumor growth (42). Chrm1 activation was also shown to suppress cancer stem cells (CSCs), which is an additional mechanism by which cholinergic signals suppress tumors (42).

The cholinergic signals also modulate the neuro-immune axis. In line with this, Dubeykovskaya et al. showed that cholinergic activation induces trefoil factor 2 (TFF2) secretion by memory T cells, which in turn suppresses MDSC to prevent colorectal cancer progression (46). Similarly, Kamia et al. showed that parasympathetic stimulation of T lymphocytes, which express Chrm1, reduces the immune checkpoint protein PD-1, leading to suppression of breast cancer (14). The cholinergic-immune axis is also essential for suppressing pancreatic cancer. For instance, cholinergic deprivation was shown to increase macrophage influx and production of TNFα, resulting in pancreatic tumor progression (47).

## Cholinergic Signals as Tumor Promoters: Molecular Mechanisms

Through selective depletion studies, Zhao et al. demonstrated that a lack of cholinergic transmission prevents gastric tumorigenesis, while Magnon et al. showed that the lack of these signals in stromal cells prevents prostate cancer metastasis (10, 11). There are multiple mechanisms that account for the tumor promoting actions of cholinergic signals. For instance, in gastric cancer cells, activated Chrm3 induces Wnt-β-catenin signals downstream of the transcriptional co-activator YAP (38). The Wnt-β-catenin signals then expand cancer stem cells (CSCs) to promote gastric tumor growth (11). Chrm3 was also shown to induce NGF in gastric cancer cells, and the NGF then acts on TrkA receptors in the nearby nerves to facilitate tumor innervation, which is an example of how cholinergic signals promote tumor axonogenesis indirectly (38). Expression of NGF and YAP was also associated with advanced stages of gastric tumors, which further substantiates the critical roles of Chrm3-NGF and Chrm3-YAP axes in tumor pathology (38). A retrospective study showed that vagotomy, and associated cholinergic deprivation, reduces gastric cancer incidence, suggesting a potential utility of cholinergic blockers in preventing gastric cancers (11). Chrm3 was also shown to promote Small Cell Lung Carcinoma (SCLC) by activating MAPK and Akt signals (40). Chrm3 promotes the invasion of cancer cells too. For instance, Chrm3 was shown to activate ERBB receptors downstream of MMP-7 in colon cancer cells, which, in turn, triggered MAPK and Akt signaling to induce cell invasion (41). Besides, inhibition of Chrm3 was shown to attenuate small intestinal neoplasia, further confirming the therapeutic utility of Chrm3 blockers in intestinal cancers (48).

In contrast to the findings by Renz et al., which showed that Chrm1 suppresses pancreatic cancer, a study by Magnon et al. showed that stromal cells-specific Chrm1 is indeed essential for prostate cancer metastasis, indicating that Chrm1 is a potential therapeutic target for prostate cancer (10, 42). A study by Coarfa et al. also supported the idea that cholinergic blockers are effective in prostate cancer by demonstrating that Botox mediated depletion of cholinergic signals improves prostate cancer outcomes (49).

**Figure 2:**
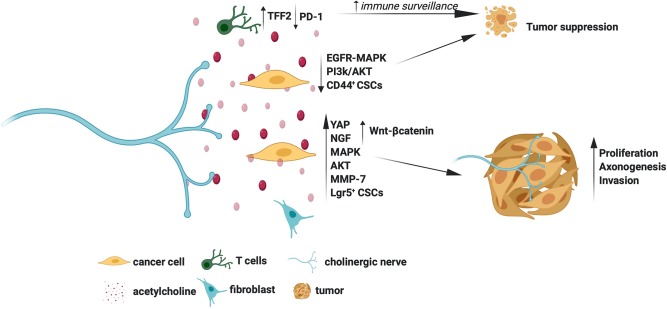
Molecular mechanisms for the tumor promoting and suppressing actions of cholinergic signals.

The molecular mechanisms by which cholinergic signals promote or suppress tumors are summarized in Figure 2. Overall, the Chrms, especially the Chrm1 and Chrm3, appear as promising targets for cancer therapy. However, it is still puzzling how the cholinergic signals elicit contrasting effects in tumors, for example, they promote and suppress gastric and pancreatic cancers, respectively (11, 38, 42). The wide expression of Chrms in cancer and stromal cells and the nerve-independent sources of acetylcholine pose hurdles in selectively studying the contribution of cholinergic nerves in tumors. Understanding of the distribution pattern of Chrms sub-types, and the various sources of acetylcholine in specific tumor types would further delineate the unique contribution of cholinergic signals in distinct tumors.

## Discussion and Future Perspectives

Accumulating evidence beyond doubt indicates that nerves offer trophic support to adult tissues. For instance, nerves maintain the adult stem cell niche, participate in wound healing, and facilitate regeneration of adult tissues (50–54). While nerves execute these functions in a controlled manner—they know when to outset and dismiss trophic secretions—it seems it is not the case when they encounter tumors, although more studies are required to make definite conclusions. No studies, however, claim that nerve-derived signals on its own are tumorigenic, but instead amplify an underlying tumor pathology (10, 11, 38, 42).

Landmark studies that addressed nerve-dependence of tumors used experimental denervation to show that lack of nerves prevents tumorigenesis (10, 11). However, the pathophysiological consequences that follow nerve damage has not been taken into account in these studies. For instance, peripheral nerve injury leads to Wallerian degeneration of nerves, recruitment of myeloid cells, and initiation of Schwann cell (SC) proliferation (55). These functional consequences may hinder experimental tumorigenesis. For instance, vagotomy was shown to induce chemokine signaling and leukocytes recruitment, similar to that occur during Wallerian degeneration, in gastric tumors (11). Denervation also induces extensive genotypic changes in healthy tissues (49). Therefore, denervation studies may be interpreted with caution when defining the mechanisms of nerve dependence of cancers.

Adult tissue function and homeostasis are maintained by coordinated actions of autonomic, sensory, and motor neurons. Therefore, a more holistic approach of considering the roles of both sensory and autonomic nerves will give additional insights into the overall effects of peripheral nerves on tumor dynamics. Sensory neurons are likely the first responders to changes in the local environment, and therefore, it is critical to understand how these neurons respond to malignant proliferation. We found that sensory neurons promote proliferation of mature macrophages and glial cells (56). Sensory nerves are also rich source of growth factors, such as NGF, BDNF, and GDNF, and interestingly, several studies demonstrated tumor-specific expression of these growth factors and their receptors (13, 38, 57). Sensory neurons also express tumor suppressor proteins and DNA repair proteins, indicating that they are equipped with tumor suppressor machinery too (58–60). Although both tumor promoting and suppressing roles are attributed to sensory neurons, more studies are required in this direction to make definite conclusions (61–63). Finally, while most studies demonstrated nerve innervation in human tumor samples, a future examination of differential distribution of sympathetic, parasympathetic, and sensory neurons may provide additional insights into whether adrenergic, cholinergic, or sensory nerve on its own is tumor permissive or suppressive.

## Author Contributions

MB participated in developing the concept, collected the literature, and composed the preliminary draft. AK formulated the concept, revised the draft extensively, composed the figures, and finalized the manuscript.

## Conflict of Interest

The authors declare that the research was conducted in the absence of any commercial or financial relationships that could be construed as a potential conflict of interest.
